# Use of serotonergic antidepressants and perioperative complications in patients undergoing lower limb arthroplasty: Systematic review and meta-analysis of comparative studies^☆^

**DOI:** 10.1016/j.jor.2025.07.009

**Published:** 2025-07-18

**Authors:** Dimitrios Grammatikopoulos, Eustathios Kenanidis, Georgios Foukarakis, Christothea-Alexandra Tsiridis, Michael Potoupnis, Eleftherios Tsiridis

**Affiliations:** a Academic Orthopaedic Department, Aristotle University Medical School, General Hospital Papageorgiou, Ring Road Efkarpia, Thessaloniki, 56403, Greece; b City St George's, University of London (Tooting), United Kingdom

**Keywords:** Serotonergic antidepressant, Total hip arthroplasty, Total knee arthroplasty, Bleeding risk, Readmission rate, Revision rate

## Abstract

**Background:**

Serotonin Reuptake Inhibitors (SRIs) are commonly utilised antidepressants among populations in Western countries; however, their use has been linked to adverse effects and postoperative complications. This systematic review assessed the risk of both early and late perioperative complications in SRIs users undergoing elective total hip arthroplasty (THA) and knee arthroplasty (TKA) compared to non-users.

**Methods:**

This study adhered to the 2020 PRISMA guidelines. A comprehensive search of PubMed, Science Direct/Scopus, and the Cochrane Database of Systematic Reviews was conducted from inception until January 2025. Comparative studies examining SRI users and non-users undergoing elective THA or TKA that addressed at least one postoperative outcome (blood loss, postoperative haemoglobin reduction, transfusion rate, length of stay, readmission and revision rate) were included. The Newcastle-Ottawa Scale was employed to evaluate the quality of the included studies. A meta-analysis was performed to assess the risk of transfusion, utilising a random-effects model.

**Results:**

Ten comparative cohort studies, including two prospective and eight retrospective studies, were included in this analysis, involving 2,098,833 patients who underwent either elective THA or TKA. Among these patients, 418,527 were using SRIs during the perioperative period, with a mean age of 65.2 years. Our meta-analysis revealed an odds ratio of 1.78 (95 % CI, 1.11, 2.83; p = 0.02) concerning the blood transfusion rate in patients utilising SRIs, although it was characterised by considerable heterogeneity (I^2^ = 97 %). The findings regarding length of stay, readmission rate, and revision rate were inconclusive.

**Conclusion:**

The use of SRIs in patients undergoing elective THA and TKA may be linked to a slightly increased risk of bleeding and need for blood transfusion. However, considering the potential benefits of these medications for this patient group, it remains uncertain whether discontinuing them during the perioperative period is advisable. More high-quality studies are required to establish an etiological relationship.

## Abbreviations:

SRIs: Serotonin Reuptake Inhibitors
SSRIs: Selective Serotonin Reuptake Inhibitors
SNRIs: Serotonin-Norepinephrine Reuptake Inhibitors
THA: total hip arthroplasty
TKA: total knee arthroplasty
PRISMA: Preferred Reporting Items for Systematic Reviews and Meta-Analyses
PICO: Population, Intervention/Exposure, Comparison, Outcome
RCTs: randomised controlled trials
LOS: length of hospital stay
NOS: Newcastle-Ottawa Scale
OR: odds ratio

## 1 Introduction

Each year, mental health issues affect millions, with many enduring these conditions for life. Estimates indicate that one in three women and one in five men will face major depression and might require medication.^1^^,^^2^ Serotonergic antidepressants, also known as Serotonin Reuptake Inhibitors (SRIs), primarily include Selective Serotonin Reuptake Inhibitors (SSRIs) and Serotonin-Norepinephrine Reuptake Inhibitors (SNRIs).^3^^,^^4^ These drugs are frequently prescribed for psychological issues such as major depressive disorder and anxiety disorders.^5^^,^^6^

Mental illness is regarded as an unreliable predictor of recovery following total hip arthroplasty (THA), with psychiatric disorders potentially heightening the risk of perioperative complications.^7^, ^8^, ^9^ Research has investigated the associations between SRI use and postoperative complications, proposing various mechanisms of drug action.^10^, ^11^, ^12^, ^13^, ^14^, ^15^, ^16^, ^17^, ^18^ Among these, the increased risk of haemorrhage and the potential necessity for blood transfusion,^19^, ^20^, ^21^ the risk of surgical site infections,^22^^,^^23^ and the risk of periprosthetic fractures due to diminished bone mineral density^24^, ^25^, ^26^ have been suggested as potential consequences of perioperative SRI use.

Patients who are on SRIs and are undergoing joint arthroplasty may face a higher risk of perioperative complications, which could negatively impact their functional outcomes and quality of life after surgery. This systematic review evaluated the effect of SRI use on outcomes after elective THA and total knee arthroplasty (TKA), assessing both early and late perioperative complications by analysing relevant comparative observational studies in the literature.

## 2 Materials and methods

This systematic review and meta-analysis were conducted in accordance with the Preferred Reporting Items for Systematic Reviews and Meta-Analyses (PRISMA 2020)^27^ and in line with the protocol agreed upon by all authors. The study was registered with the PROSPERO database of systematic reviews (CRD42023473653).

### 2.1 Search strategy

A comprehensive search of all databases in PubMed, Science Direct/Scopus, and the Cochrane Database of Systematic Reviews was performed from inception until January 2025 to identify studies reporting outcomes for patients undergoing THA/TKA who were also receiving SRIs. Furthermore, the reference lists of relevant published articles were manually searched to identify any missing records. The exact search string that was used is presented in.Supplementary Appendix 1.

### 2.2 Eligibility criteria

The following PICO (Population, Intervention/Exposure, Comparison, Outcome) criteria were used for inclusion in this meta-analysis: (i) population: patients undergoing elective THA or TKA; (ii) intervention/exposure: perioperative SRI use; (iii) comparison group: patients undergoing THA or TKA who are not utilising SRIs; (iv) outcome: postoperative complications.

The following inclusion criteria were established: (i) randomised controlled trials (RCTs) and non-RCTs, prospective and retrospective cohort and case-control studies; (ii) studies involving adult patients (>18 years) who were using serotonergic antidepressants and underwent elective THA or TKA; (iii) studies assessing at least one of the following outcomes: bleeding risk (blood loss, postoperative haemoglobin reduction, transfusion rates), length of hospital stay (LOS), readmission rates, and need for revision surgery; (iv) studies yielding extractable data; (v) studies conducted in English.

The following exclusion criteria were also implemented: (i) studies involving patients who underwent non-elective THA, hip hemiarthroplasty, or other than the hip/knee arthroplasty; (ii) review articles, case series, case reports, letters to the editor, editorial comments; (iii) cadaveric or animal studies; (iv) studies published in a language other than English.

### 2.3 Study selection

**Fig. 1:**
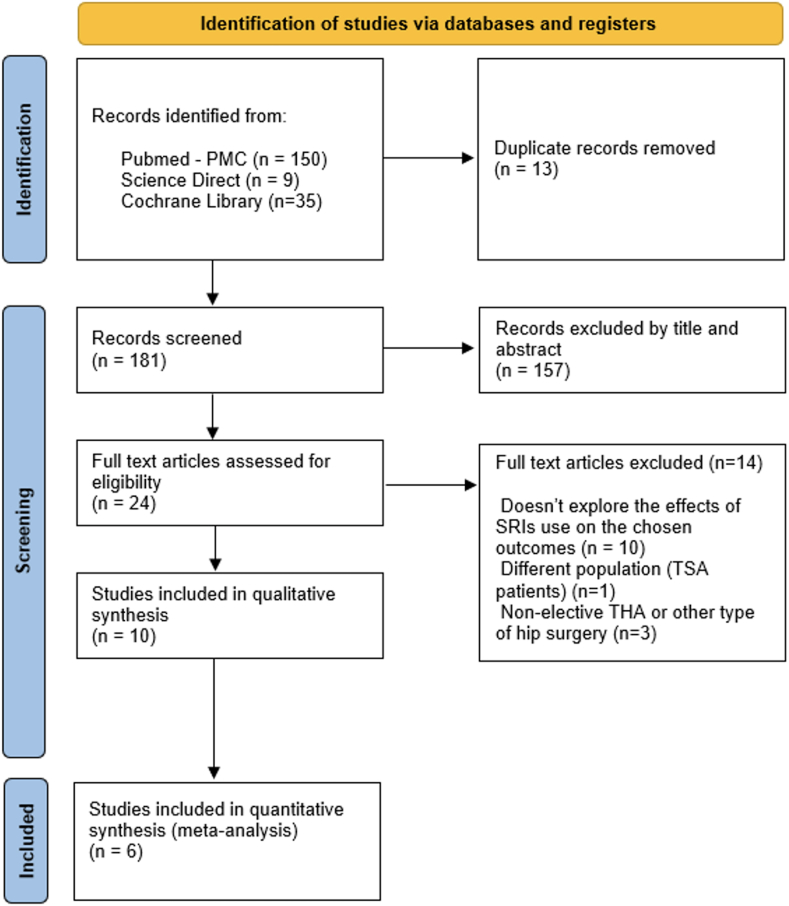
Preferred Reporting Items for Systematic Reviews and Meta-analyses (PRISMA) flowchart (***SRIs:****Serotonin Reuptake Inhibitors,****TSA:****Total Shoulder Arthroplasty,****THA:****Total Hip Arthroplasty)*.

Two authors, D.G. and G.F., independently conducted the literature review and screened the results, identifying relevant studies according to predefined eligibility criteria. Any discrepancies between the two authors were resolved through discussion with a third senior author (E.K.). A PRISMA flowchart illustrating the study selection process is provided (Fig. 1).

### 2.4 Data extraction and analyses

The same authors independently reviewed the selected studies and extracted the following data: (1) study type, authorship, publication year, country, and study design; (2) study population, comprising total sample and subgroup sizes, surgery time, and patient demographics; (3) blood loss, postoperative haemoglobin decline, transfusion rate, LOS, readmission rate, and need for revision surgery.

Meta-analysis was performed using Review Manager (RevMan) Version 5.4. The Cochrane Collaboration, 2020.^28^

### 2.5 Risk of Bias and study quality assessment

The Newcastle-Ottawa Scale (NOS) was utilised to assess the methodological quality of these cohort studies. Discrepancies were resolved by consensus after consulting the senior author (E.K.). The NOS evaluates non-randomised studies in meta-analyses using a star system that awards up to nine stars per study. A maximum of four stars can be earned in selection, two in comparability, and three in exposure/outcome. Scores of 0–3 denote “Low quality,” 4–6 indicate “Moderate quality,” and 7–9 signify “High quality.” The quality assessment outcomes are summarised in Supplementary Appendix 2 and 3.

## 3 Results

### 3.1 Search results

The initial search yielded 194 studies. After removing duplicates, 181 were screened based on titles and abstracts. Of these, 24 were suitable for a comprehensive review. Ultimately, 10 studies were selected for analysis in this systematic review.^29^, ^30^, ^31^, ^32^, ^33^, ^34^, ^35^, ^36^, ^37^, ^38^ The screening process is illustrated in Fig. 1 (see Table 1).

### 3.2 Included studies design & patients’ demographics

The analysis included studies published from 2010 to 2024: eight retrospective^29^^,^^30^^,^^32^, ^33^, ^34^, ^35^, ^36^^,^^38^ and two prospective cohorts.^31^^,^^37^ Five studies were from the United States,^30^^,^^33^^,^^34^^,^^36^^,^^38^ three from Denmark,^31^^,^^35^^,^^37^ one from Canada,^29^ and one from the Netherlands.^32^ Six studies included both THA and TKA patients^29^, ^30^, ^31^^,^^33^^,^^34^^,^^37^; two focused on THA,^32^^,^^35^ and two solely on TKA.^36^^,^^38^ (Table 2) A total of 2,098,833 patients undergoing either elective THA or TKA were analysed. Among them, 418,527 individuals used SRIs during the perioperative period, comprising the studied cohorts, while the rest served as controls. The average age of SRI users was 65.2 years, compared to 65.4 years for the control group. The percentage of male patients was 30.4 % for SRI users and 41.5 % for controls.

**Table 1:** Demographics, patients’ characteristics and study design of the included studies.

Author	Year	Study Type	Total Number of Patients (THAs/TKAs)	SRI group Patients (THAs/TKAs)	Control group Patients (THAs/TKAs)	Time of surgery	Age at surgery SRI/control (years)	Follow up	Sex SRI-Control (men/women)	Studied Outcomes
Bourget-Murray et al.^29^	2022	RCS	28386 (10953/17433)	2303 (851/1452)	26083 (10102/15981)	2014–7	65.7/66.3	12 months	577/1726–11350/14733	Transfusion rate, Length of stay, Readmission rate
Tavakoli et al.^30^	2012	RCS	194 (104/90)	71 (34/37)	123 (70/53)	2005–11	55.4/56.2	3 days^a^	21/50 – 37/86	Post-op Hgb drop, Blood loss, Transfusion rate
Gylvin et al.^31^	2017	PCS	8402 (4423/3979)	569 (276/293)	7833 (4147/3686)	2010–12	69.2/67.5	N/A^a^	152/417–3435/4398	Transfusion rate
van Haelst et al.^32^	2010	RCS	365 (365/0)	66 (66/0)	285 (285/0)	2004–8	67/68	N/A^a^	15/51–93/192	Blood loss, Transfusion rate
Belay et al.^33^	2019	RCS	10069 (4485/5584)	1900 (740/1160)	8169 (4424/3745)	2013–17	65/66.1	N/A^a^	485\1415–3745\4424	Post-op Hgb drop, Blood loss, Transfusion rate, Length of stay
Yao et al.^34^	2018	RCS	20112 (9666/10446)	2186 (NS)	17926 (NS)	2002–9	66/66	>10 years	715/1471–8422/9504	Revision rate
Dall et al.^35^	2014	RCS	1318 (1318/0)	83 (83/0)	1202 (1202/0)	2007–12	65/65	N/A^a^	32/51 – 635/567	Blood loss, Transfusion rate
Kuyl et al.^36^	2024	RCS	974844 (0/974844)	201562 (0/201562)	773282 (0/773282)	2010–22	65/66	2+ years	63694/137868–320905/452377	Revision rate
Jørgensen et al.^37^	2015	PCS	8757 (4597/4160)	467 (NS)	7517 (ΝS)	2010–12	69/68	3 months	3714/5043 (all patients)	Length of stay, Readmission rate
Ratnasamy et al.^38^	2024	RCS	1046386 (0/1046386)	209320 (0/209320)	837066 (0/837066)	Until 2020	65.2/65.3	5+ years	57977/151343–231826/605240	Revision rate, Transfusion rate, Readmission rate

THA: Total Hip Arthroplasty, TKA: Total Knee Arthroplasty, SRI: Serotonin Reuptake Inhibitor, RCS: Retrospective Cohort Study, PCS: Prospective Cohort Study, N/A: Not Available, NS: Non-Specified.

a Until discharge from hospital.

**Table 2:** Operative and implant characteristics, and functional scores of the included studies.

Author	Type of operation	Surgical approach	Type of anesthesia (SRI/Control)	Type of fixation	Type of implant	Functional score SRI-Control (preop/12 months postop)	
Bourget-Murray et al.^29^	THA & TKA	Various	N/A	N/A	N/A	***THA*** **WOMAC** 36.3/39.1–76.2/81.6 **EQ-5D-5L** 0.37/0.43–0.74/0.81	**TKA** **WOMAC** 41.8/45.6–73.2/77.9 **EQ-5D-5L** 0.46/0.53–0.74/0.79
Tavakoli et al.^30^	THA & TKA	N/A	N/A	N/A	N/A	N/A	
Gylvin et al.^31^	THA & TKA	N/A	N/A	N/A	N/A	N/A	
van Haelst et al.^32^	THA	N/A	Spinal 62 % /Spinal = 71 %	SRI: Cem 62 % Control: Cem 51 %	N/A	N/A	
Belay et al.^33^	THA & TKA	N/A	N/A	N/A	N/A	N/A	
Yao et al.^34^	THA & TKA	N/A	N/A	THA: Unc cup = 86 % TKA: Cem = 97.6 %	N/A	N/A	
Dall et al.^35^	THA	Posterolateral	N/A	THA: 100 % Unc	Stems: Bimetric® (Biomet) Corail® (DePuy) Cups: Trilogy® (Zimmer) Mallory® (Biomet) Exceed® (Biomet)	N/A	
Kuyl et al.^36^	TKA	N/A	N/A	N/A	N/A	N/A	
Jørgensen et al.^37^	THA & TKA	N/A	N/A	N/A	N/A	N/A	
Ratnasamy et al.^38^	TKA	N/A	N/A	N/A	N/A	N/A	

THA: Total Hip Arthroplasty, TKA: Total Knee Arthroplasty, N/A: Not Available, WOMAC: Western Ontario and McMaster Universities Osteoarthritis Index, EQ-5D-5L: Cem: Cemented, Unc: Uncemented.

### 3.3 Studied outcomes and follow-up

Seven studies included at least one parameter related to postoperative bleeding.^29^, ^30^, ^31^, ^32^, ^33^^,^^35^^,^^38^ All studies assessed transfusion rates; four examined estimated blood loss,^30^^,^^32^^,^^33^^,^^35^ and two postoperative haemoglobin drop.^30^^,^^33^ Other outcomes included LOS (three studies),^29^^,^^33^^,^^37^ readmission rate (three studies),^29^^,^^37^^,^^38^ and need for revision (three studies).^34^^,^^36^^,^^38^ Follow-up varied by study objectives. Five studies focused on postoperative bleeding risk, limiting follow-up to the immediate period.^30^, ^31^, ^32^, ^33^^,^^35^ The other five explored long-term outcomes, with follow-up ranging from 3 months to over 10 years.^29^^,^^34^^,^^36^, ^37^, ^38^ (Table 2)

### 3.4 Operative and implant characteristics

Surgical data were limited in most studies. Two studies examined the THA surgical approach,^29^^,^^35^ while one provided information on anesthesia type.^32^ Three studies reported on fixation methods. The first study found that 62 % of SRI users had cemented femoral stem fixation compared to 71 % of controls.^32^ The second reported that 86 % of all THA patients received uncemented acetabular component fixation, with a 97.6 % rate of cemented fixation for either femoral and/or tibial components in TKA patients.^34^ The third study noted that 100 % of THA operations used uncemented component fixation,^35^ and it was the only one to detail THA components. Finally, one study evaluated patients’ functionality and health-related quality of life^29^ with a similar mean increase at 12 months for both groups (Table 3).

**Table 3:** Postoperative complications of SRI patients vs control group in the included studies.

Author	Complications					
	Perioperative bleeding			Length of stay (days)	Readmission rate	Need for revision
	Estimated blood loss (mL)	Postoperative Hgb drop (day 1) mg/dl	Transfusion rate (%)			
Bourget-Murray et al.^29^	N/A	N/A	THA = 11.16/5.45 TKA = 5.1/3.79	THA = 4.36/3.67 TKA = 3.98/3.62	THA = 4.35 %/3.2 % TKA = 4.61 %/3.05 % at 30 days	N/A
Tavakoli et al.^30^	255/230	2.7/5.5	0	N/A	N/A	N/A
Gylvin et al.^31^	N/A	N/A	19.2/8.9	N/A	N/A	N/A
van Haelst et al.^32^	672/566	N/A	26/19	N/A	N/A	N/A
Belay et al.^33^	817/811	2.3/2.3	THA = 8.5/4.8 TKA = 3.4/3.1	2.3/2.3	N/A	N/A
Yao et al.^34^	N/A	N/A	N/A	N/A	N/A	0.77 HR at avg. 6 years
Dall et al.^35^	400/350	N/A	N/A	N/A	N/A	N/A
Kuyl et al.^36^	N/A	N/A	N/A	N/A	N/A	2.4 %/1.6 % at 2 years
Jørgensen et al.^37^	N/A	N/A	N/A	OR for LOS>4 days = 2.19	OR for Readmission at 30 days = 1.97 90 days = 1.77	N/A
Ratnasamy et al.^38^	N/A	N/A	2.3/2.5	N/A	6.7 %/7.3 % (TKA) at 90 days	3.1 %/3.6 % at 5 years

THA: Total Hip Arthroplasty, TKA: Total Knee Arthroplasty, N/A: Not Available, HR: Hazard Ratio, avg.: average, OR: Odds Ratio, LOS: Length of Stay.

### 3.5 Patients’ pharmacological data

All patients in the cohort groups of the studies were receiving treatment with at least one serotonergic antidepressant medication at the time of surgery, including SSRIs, SNRIs, or any other antidepressant classified as “serotonergic” due to its high affinity for the serotonin reuptake transporter, or a combination of these. Table 4 provides detailed information on the drug types used in both the cohort and control groups of the included studies.

**Table 4:** Pharmacological data of the studied cohorts and the control groups in the included studies.

Author	Serotonergic antidepressants used by the studied cohort	Antidepressant medication used by the control group
Bourget-Murray et al.^29^	SSRIs only	No ADs or NS non-SSRI ADs
Tavakoli et al.^30^	SSRIs: 43, SNRIs:24, other SRIs (Trazadone = 3, Mirtazapine = 1)	No ADs or NS non-SSRI ADs
Gylvin et al.^31^	SSRIs: 466, SSRIs plus other NS ADs:103	No ADs
van Haelst et al.^32^	SSRIs: 55, SNRIs: 11	No ADs^a^
Belay et al.^33^	SSRIs: 1640, SSRIs plus other NS ADs: 260	No ADs
Yao et al.^34^	SSRIs: 1536, SSRIs plus other NS ADs: 650	No ADs or NS non-SSRI ADs
Dall et al.^35^	SSRIs only, SNRIs only or Clomipramine only	No ADs^a^
Kuyl et al.^36^	SSRIs	No ADs or NS non-SSRI ADs
Jørgensen et al.^37^	SSRIs:390, SSRIs plus other NS ADs: 72	No ADs
Ratnasamy et al.^38^	SSRIs or SNRIs	No ADs

SSRI: Selective Serotonin Reuptake Inhibitor, AD: Antidepressant, NS: Non-Specified, SNRI: Serotonin Norepinephrine Reuptake Inhibitor.

a The study included a second cohort of non-SRI antidepressant users.

### 3.6 Outcomes

#### 3.6.1 Perioperative bleeding

Seven studies^29^, ^30^, ^31^, ^32^, ^33^^,^^35^^,^^38^ assessed perioperative bleeding risk using these methodologies:

1. Estimated blood loss during surgery
2. Change in haemoglobin levels on the first postoperative day compared to preoperative levels.
3. Proportion of patients receiving blood transfusions.

All seven studies reported transfusion rates. Two compared estimated blood loss,^32^^,^^35^ and two evaluated all three parameters.^30^^,^^33^

**Fig. 2:**
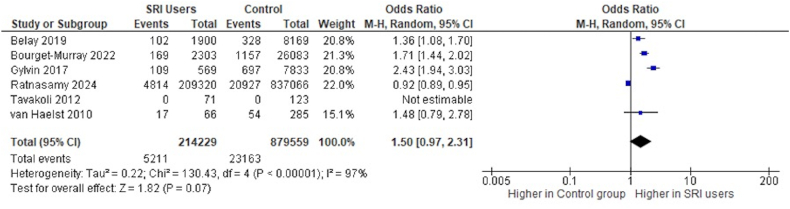
Forest Plot for comparison of transfusion rates between SRI users and control group patients.

We conducted a meta-analysis on transfusion rates using six of seven studies.^29^, ^30^, ^31^, ^32^, ^33^^,^^38^ The study by Dall et al.^35^ lacked specific transfusion rates for each group and was excluded. However, it noted no significant difference between groups. Due to variability, we employed a random-effects model. Four studies showed significantly higher transfusion rates for SRI users,^29^^,^^31^, ^32^, ^33^ while two showed no significant difference.^30^^,^^38^ Our meta-analysis indicated an odds ratio (OR) of 1.78 (95 % CI, 1.11–2.83; p = 0.02) for blood transfusions in SRI users. The heterogeneity was substantial (I^2^ = 97 %) and statistically significant (Fig. 2).

The studies reporting estimated blood loss and haemoglobin reduction lacked adequate data for meta-analysis. However, three of four studies showed a statistically significant increase in blood loss among SRI users.^32^^,^^33^^,^^35^ (Table 3) Tavakoli et al.^30^ found no significant differences in estimated intraoperative blood loss or postoperative haemoglobin levels, but since non-users exhibited higher preoperative haemoglobin levels, a greater decrease was noted among this group (5.5 vs 2.7 mg/dL for SRI users). Conversely, Belay et al.^33^ noted no haemoglobin reduction difference between groups (2.3 mg/dL for both), with non-users having significantly higher preoperative haemoglobin levels.

For the remaining outcomes—LOS, readmission rate, and revision rate—a quantitative synthesis was not performed due to the limited number of studies providing data for each and the absence of necessary statistical measures in some studies.

#### 3.6.2 LOS

Three studies examined SRI usage's impact on LOS.^29^^,^^33^^,^^37^ Belay et al.^33^ found no significant difference in LOS between SRI users and non-users, both averaging 2.3 days. In contrast, Bourget-Murray et al.^29^ reported a longer LOS for SRI users undergoing THA and TKA, with averages of 4.2 days for SRI users and 3.6 days for controls. Jørgensen et al.^37^ classified LOS over 4 days as “prolonged” and noted a correlation between SRI usage and increased risk of prolonged stay (OR: 2.19; 1.62 to 2.97).

#### 3.6.3 Readmission rate

Three studies assessed readmission risk due to postoperative complications after hospital discharge, evaluated at either 30 or 90 days post-surgery, or both.^29^^,^^37^^,^^38^ Bourget-Murray et al.^29^ reported a 30-day readmission rate, with SRI users having a higher rate than controls (4.35 vs 3.2 % for THA, 4.61 vs 3.05 % for TKA). Jørgensen et al.^37^ found increased readmission risk for SRI users at 30 days (OR: 1.97; 1.38 to 2.82) and 90 days (OR: 1.77; 1.29 to 2.43). In contrast, Ratnasamy et al.^38^ reported a lower 90-day readmission rate for SRI users compared to non-users (6.7 vs 7.3 %), despite SRI users facing higher risk for most short-term adverse events measured in that study.

#### 3.6.4 Revision rate

Three studies with long-term follow-up evaluated revision surgery risks.^34^^,^^36^^,^^38^ Each used different endpoints at 2, 5, and an average of 6 years. Kuyl et al.^36^ reported a higher all-cause revision rate for SRI users compared to non-users at 2 years (2.4 vs 1.6 %). Ratnasamy et al.^38^ found SRI use correlated with a slightly lower revision rate at 5 years (3.1 vs 3.6 %). Yao et al.^34^ indicated a reduced risk for all-cause revision at an average of 6 years (hazard ratio: 0.77; 95 % CI, 0.61–0.96; p = 0.001). Notably, Kuyl and Ratnasamy's studies focused on TKA patients, while Yao's included THA and TKA patients.

## 4 Discussion

This systematic review evaluated the association between serotonergic antidepressant use and the incidence of potential perioperative complications in patients undergoing elective THA and TKA. Our findings indicate that users might face a modestly increased risk of complications during the immediate postoperative period, including the need for transfusion due to bleeding. However, the results were inconclusive regarding other short-term complications, such as prolonged LOS, as well as long-term complications, including readmission and revision.

Our meta-analysis found a significantly higher risk of transfusion for patients who utilised SRIs compared to the control group, marked by substantial heterogeneity. Notably, the study by Ratnasamy et al.,^38^ the largest in population size, reported a slightly reduced transfusion risk for users, but was not weighted accordingly due to our random-effects model. Additionally, in the absence of universal blood transfusion guidelines for THA and TKA, criteria differ significantly among institutions.^39^ Only one study, by Belay et al.,^33^ specified blood transfusion criteria.

Blood loss was greater for SRI users in all four studies,^30^^,^^32^^,^^33^^,^^35^ with a significant increase in three.^32^^,^^33^^,^^35^ This indicates likely reduced hemostasis during surgery but is not a highly reliable measure.^40^ The increased bleeding risk from SRIs may stem from decreased intraplatelet serotonin concentration, affecting platelet aggregation.^13^, ^14^, ^15^, ^16^ Furthermore, major surgeries correlate with lower platelet serotonin and higher plasma serotonin levels.^20^^,^^41^ These effects may synergistically impair hemostasis, increasing bleeding risk in surgical patients on SRIs. However, studies on postoperative haemoglobin levels showed similar results in both groups,^30^^,^^33^ questioning the clinical significance of this finding.

The LOS increased in two of three studies,^29^^,^^37^ which also showed a higher readmission risk at 30 and 90 days. This may relate to more minor short- and medium-term adverse events in SRI patients, as noted by Ratnasamy et al.^38^ However, postoperative hospitalisation, discharge, and readmission protocols vary by institution, making comparisons of absolute values impractical.^42^ Jorgensen et al.^37^ provided data on readmission causes, distinguishing between surgery-related and non-surgery-related reasons. SRI users had a higher rate of surgery-related readmissions, significantly for falls, but not for infections or hip dislocations.

The all-cause revision rate results were inconsistent. One study indicated a higher revision risk among SRI users at two years,^36^ while two studies with longer follow-ups^34^^,^^38^ reported a lower risk for SRI users at five and six years. The varying onset timelines for causes necessitating revision THA and TKA surgery may clarify this. It suggests that, despite the link between SRI use and decreased bone mineral density, this use likely does not compromise long-term implant survival. Additionally, treating underlying depression could be a protective factor.^60,61^ However, research by Yao et al.^34^ showed SSRI users had a significantly lower revision risk compared to other antidepressants, suggesting a possible true biologic effect tied to SSRIs. Nonetheless, despite conflicting results, all studies concluded that there is insufficient evidence to establish a causal relationship.

Our study has limitations. Although all included studies scored high on the Newcastle-Ottawa Quality Control Assessment Scale, they were non-randomized cohort studies. Only two were prospective; the rest were retrospective, resulting in a low level of evidence. Study designs varied significantly, with most covering a subset of outcomes and none addressing all comprehensively. Population sizes and compositions differed markedly. While average patient age and male-to-female ratios were similar, six studies included THA and TKA patients in varying ratios. In contrast, two studies focused solely on THA patients, and two on TKA patients. Notably, THA and TKA have different complication profiles, and the use of tourniquets during TKA adds complexity.^43^^,^^44^ In most studies, details about surgical operations-like anesthesia type, surgical approach, fixation method, implant type, and tourniquet use for TKA-were scarce or absent. This omission is crucial, as these factors could influence our review outcomes.^45^^,^^46^ This finding underscores that included studies reported outcomes without a link to this specific medication.

The variation among studies in pharmacological profiles of cohorts and control groups was noteworthy. All cohort patients were on at least one “serotonergic” antidepressant, but SSRIs, SNRIs, Trazodone, Mirtazapine, and Clomipramine belong to different subclasses. Even within SSRIs and SNRIs, a wide range of agents were used, differing significantly across studies. While these medications have a similar mechanism through high affinity for the serotonin reuptake transporter, it raises concerns about linking adverse events to specific effects. Although authors excluded patients using drugs that increase postoperative risks, many patients likely used other unaccounted medications, impacting results. Moreover, six studies^31^, ^32^, ^33^^,^^35^^,^^37^^,^^38^ had control groups of patients not taking any antidepressants, while four^29^^,^^30^^,^^34^^,^^36^ had varying percentages using unspecified non-SSRI antidepressants. Additionally, data on SRI dosage was absent in the studies. A retrospective study by Öndemark et al. found a dose-dependent increase in the risk of postpartum bleeding with SSRIs during delivery,^47^ suggesting a potential dose-response relationship that may affect complication rates but cannot be fully evaluated.

The distinction between depression as a medical condition and its pharmacological treatment is significant. Depression is a considerable risk factor for adverse postoperative outcomes after THA and TKA, including increased bleeding, longer hospital stays, and higher readmission rates.^48^^,^^49^ This complicates differentiating the effects of depression's pathophysiological processes from those of the serotonergic agents used by these patients. Some study findings may relate to the disease itself rather than being adverse outcomes of the medications. Recent systematic reviews suggest that effective management of depression, particularly with SRIs, is linked to improved postoperative outcomes and a reduced risk of long-term complications.^50^^,^^51^

A limitation of our review was that most included studies lacked data on the medical histories of the populations, especially regarding the absence of an official depression diagnosis for both cohort and control groups. A notable percentage of cohort patients might have been using SRIs for non-depression conditions or not showing any symptoms of depression at surgery. Conversely, some control group patients, particularly those not on antidepressants, may have had undiagnosed or untreated depression.

## 5 Conclusions

In conclusion, our review indicates that using SRIs in elective THA and TKA patients may slightly increase bleeding risk and postoperative blood transfusions. This complication, along with other short-term adverse events, might marginally extend length of stay and readmission risk. However, it remains unclear if discontinuing these medications during the perioperative period is advisable. Long-term risks of all-purpose revision are inconsistent; although some mechanisms are proposed, no clear etiological relationship has been shown. To clarify these issues and better understand the pathophysiologic changes in SRI users, high-quality randomised control trials and prospective cohort studies with extensive follow-up are needed.

## CRediT authorship contribution statement

**Dimitrios Grammatikopoulos:** Writing – review & editing, Data curation, Formal analysis, Methodology, Writing – original draft. **Eustathios Kenanidis:** Writing – review & editing, Data curation, Formal analysis, Methodology, Validation, Supervision, Conceptualization. **Georgios Foukarakis:** Conceptualization, Writing – review & editing. **Christothea-Alexandra Tsiridis:** Writing – review & editing, Writing – original draft. **Michael Potoupnis:** Writing – review & editing, Supervision. **Eleftherios Tsiridis:** Writing – review & editing, Supervision, Conceptualization.

## Informed consent

This study is a systematic review, so informed consent was not obtained. The study was registered with the PROSPERO database of systematic reviews (CRD42023473653).

## Ethics statement

This study is a systematic review. Ethics approval was waived for this study because no patients’ data were reported. The study was registered with the PROSPERO database of systematic reviews (CRD42023473653).

## Funding

This research did not receive any specific grant from funding agencies in the public, commercial, or not-for-profit sectors.

## Declarations of interest

None.
