# Supplementary material for: Use of serotonergic antidepressants and perioperative complications in patients undergoing lower limb arthroplasty: Systematic review and meta-analysis of comparative studies
Source: J Orthop. 2025 Jul 18;67:318–25. doi: 10.1016/j.jor.2025.07.009 (PMC12304700; doi:10.1016/j.jor.2025.07.009)
Supplement: Multimedia component 1 [file mmc1.docx]

| **Appendix 3.**  **NEWCASTLE – OTTAWA QUALITY CONTROL ASSESSMENT SCALE** | | | | |
| --- | --- | --- | --- | --- |
| **Study** | **Selection** | **Comparability** | **Outcome** | **TOTAL** |
| Bourget-Murray et al. | 1=a 2=a  3=a 4=a  Total=4* | a+b  Total=2* | 1=b  2=a  3=a  Total=3* | 9* |
| Tavakoli et al. | 1=a 2=a  3=a 4=a  Total=4* | a+b  Total=2* | 1=b  2=a  3=a  Total=3* | 9* |
| Gylvin et al. | 1=b 2=a  3=a 4=a  Total=4* | a+b  Total=2* | 1=b  2=a  3=a  Total=3* | 9* |
| van Haelst et al. | 1=a 2=a  3=a 4=a  Total=4* | a+b  Total=2* | 1=b  2=a  3=a  Total=3* | 9* |
| Belay et al. | 1=a 2=a  3=a 4=a  Total=4* | a+b  Total=2* | 1=b  2=a  3=a  Total=3* | 9* |
| Yao et al. | 1=b 2=a  3=a 4=a  Total=4* | a+b  Total=2* | 1=b  2=a  3=b  Total=3* | 9* |
| Dall et al. | 1=b 2=a  3=a 4=a  Total=4* | a+b  Total=2* | 1=b  2=a  3=a  Total=3* | 9* |
| Kuyl et al. | 1=b 2=a  3=a 4=a  Total=4* | a+b  Total=2* | 1=b  2=a  3=b  Total=3* | 9* |
| Jørgensen et al. | 1=a 2=a  3=a 4=a  Total=4* | a+b  Total=2* | 1=b  2=a  3=a  Total=3* | 9* |
| Ratnasamy et al. | 1=a 2=a  3=a 4=a  Total=4* | a+b  Total=2* | 1=b  2=a  3=a  Total=3* | 9* |
| ***Appendix 3.*** *Quality assessment of included studies* | | | | |
