# Supplementary material for: Use of serotonergic antidepressants and perioperative complications in patients undergoing lower limb arthroplasty: Systematic review and meta-analysis of comparative studies
Source: J Orthop. 2025 Jul 18;67:318–25. doi: 10.1016/j.jor.2025.07.009 (PMC12304700; doi:10.1016/j.jor.2025.07.009)
Supplement: Multimedia component 2 [file mmc2.docx]

**Appendix *2*.**
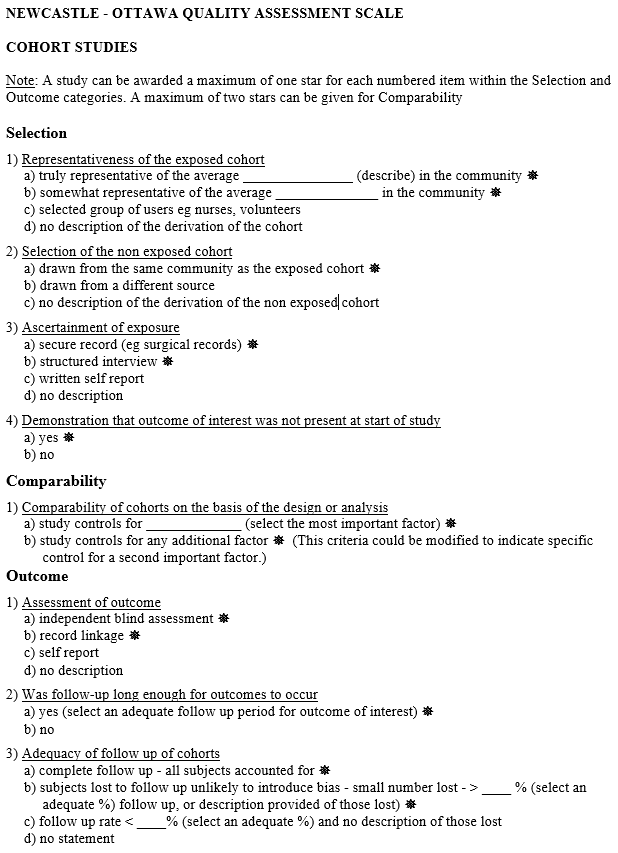


**Appendix 2.** The Newcastle-Ottawa Scale (NOS) for assessing the quality of nonrandomised studies in meta-analyses - Manual
