# Supplementary material for: Use of serotonergic antidepressants and perioperative complications in patients undergoing lower limb arthroplasty: Systematic review and meta-analysis of comparative studies
Source: J Orthop. 2025 Jul 18;67:318–25. doi: 10.1016/j.jor.2025.07.009 (PMC12304700; doi:10.1016/j.jor.2025.07.009)
Supplement: Multimedia component 3 [file mmc3.docx]

The following search terms [All Fields], along with the appropriate MeSH terms, while employing Boolean operators, were used: *"SRI" OR "SRIs" OR "SSRI*" OR "serotonergic antidepressant*" OR "serotonergic*"OR "Selective Serotonin Reuptake Inhibitor*" OR "Serotonin–norepinephrine reuptake inhibitor*" OR "Serotonin–noradrenaline reuptake inhibitor" OR "Serotonin and norepinephrine reuptake inhibitor*" OR "Serotonin and noradrenaline reuptake inhibitor*"OR "Selective Serotonin Reuptake Inhibitors" [Pharmacological Action] OR "Selective Serotonin Reuptake Inhibitors"[Mesh]OR "Selective Serotonin Reuptake Inhibitors/pharmacology"[Mesh] OR "Selective Serotonin Reuptake Inhibitors/adverse effects" OR "Serotonin and Noradrenaline Reuptake Inhibitors"[Pharmacological Action]OR "Serotonin and Noradrenaline Reuptake Inhibitors"[Mesh] OR "Serotonin and Noradrenaline Reuptake Inhibitors/pharmacology"[Mesh] OR "Serotonin and Noradrenaline Reuptake Inhibitors/adverse effects" AND "hip arthroplast*" OR "hip replacement*" OR "knee arthroplast*" OR "knee replacement*" OR "joint arthroplast*" OR "joint replacement*" OR "arthroplast*" OR "Arthroplasty, Replacement, Hip"[Mesh] OR "Arthroplasty, Replacement, Knee"[Mesh].*
